# Availability and implementation of guidelines in European child primary health care: how can we improve?

**DOI:** 10.1093/eurpub/ckac114

**Published:** 2022-08-25

**Authors:** Paul L Kocken, Nicole M C van Kesteren, Renate van Zoonen, Sijmen A Reijneveld

**Affiliations:** Department of Psychology, Education and Child Studies, Erasmus School of Social and Behavioural Sciences, Erasmus University Rotterdam, Rotterdam, The Netherlands; Department of Child Health, TNO, Leiden, The Netherlands; Department of Child Health, TNO, Leiden, The Netherlands; Department of Health Sciences, University Medical Center Groningen, University of Groningen, Groningen, The Netherlands

## Abstract

**Background:**

Clinical guidelines are important for providing high-quality child primary health care. We aimed to assess the availability, use and achieved delivery of guidelines in the European Union (EU).

**Methods:**

We used a case study design to ascertain expert views on guidelines in six countries representing the EU. The experts completed an online questionnaire (response 49%), asking about their perception of guideline availability and implementation regarding three topics that represent prevention and care, i.e. vaccination, assessment of mental health and asthma care.

**Results:**

According to the respondents all countries had guidelines available for asthma care. For vaccination and mental health assessment respondents agreed to a lesser degree that guidelines were available. Implementation of guidelines for vaccination was mostly perceived as intended, but implementation of the guidelines for mental health assessment and asthma care was limited. Notable barriers were complexity of performance, and lack of training of professionals and of financial resources. Important facilitators for guideline implementation were the fit with routine practice, knowledge and skills of professionals and policy support. We found no clear relationship of guideline availability and implementation with type of child primary health care system of countries, but strong governance and sufficient financial resources seemed important for guideline availability.

**Conclusions:**

Availability and implementation of clinical guidelines in child primary health care vary between EU countries. Implementation conditions can be strongly improved by adequate training of professionals, stronger governance and sufficient financial resources as facilitating factors. This can yield major gains in child health across Europe.

## Introduction

The importance of high-quality primary care is vital, as it regards the first point of contact with health problems of children and young people.^1–3^ A poor performance on quality indicators such as governance, accessibility and continuity of care by child primary health care in the European Union (EU), may be detrimental for the health outcomes of its young inhabitants.^4^

Quality of care can be improved using clinical guidelines, which contribute to compliance with best care practices and to standardized delivery of care.^5^ This, e.g. holds for guidelines on monitoring of asthma in children, issued by professional organizations, such as the European Respiratory Society and the Global Initiative for Asthma.^6–9^ To affect outcomes in patients, guidelines need to be adopted or used by the professional health care workers, and carefully implemented with special attention to the achieved delivery in the intended way.^10–14^ Literature on the quality of guidelines in treatment of paediatric diseases noted for instance tensions in the applicability of guidelines with lack of attention to facilitators and barriers of implementation.^15–17^ Moreover, the use and delivery of guidelines is likely to differ between EU countries due to system factors which relate to implementation theory, such as the varying compatibility with values and daily practice of local care professionals, and the organizational and socio-political context.^18^^,^^19^

Evidence is lacking on the implementation of guidelines in the practice of Europe’s child primary health care systems, characterized by the lead professional, i.e. General Practitioner (GP) or Paediatrician, and regulation of access to specialized services.^4^^,^^20^ We therefore aimed to assess the availability, use and achieved delivery of three guidelines regarding vaccination, mental health assessment and asthma care, and the factors affecting their implementation across the EU. The study was part of the Models of Child Health Appraised (MOCHA) project that systematized and appraised the types of models of child primary health care in all 30 EU/EEA countries (http://www.childhealthservicemodels.eu/ 22 August 2022, date last accessed).^4^

## Methods

We used a case study design to ascertain multiple stakeholders’ perspectives on the topics of study, i.e. availability, use and delivery of guidelines.^21^^,^^22^

### Selection of guidelines

We selected guidelines on three differing topics based on the following criteria that they: 1. were pivotal for child health in the European context, 2. covered the different stages of childhood from early age to adolescence, 3. represented the full range of the functions prevention, surveillance and diagnosis in primary care, including somatic and mental health care. Experts working in the MOCHA project eventually selected: (i) vaccination of children, (ii) identification or assessment of mental health problems in adolescents aged 10–18 years, and (iii) asthma care in children aged 6–18 years. In preparing our study we searched for international accepted standards and guidelines on the three selected topics, using literature and consultation of experts. Guidelines could also be formal procedures, laid down in documents by governmental bodies or expertise centres, to leave open the inclusion of such formally authorized procedures, e.g. vaccination schemes. We denote further in the text these formal procedures also as ‘guidelines’.

### Two-stage sampling: countries and experts within countries

Experts were sampled following a two-stage procedure. First, we selected six countries that were exemplary of the types of primary care systems in the EU based on variation in: (i) the lead practitioner who is responsible for the primary care for the child, and (ii) the way the system can be accessed by the patient, i.e. gatekeeper or open access systems.^4^ Combining these two characteristics led to the following types of country systems: (a) gatekeeper-GP led: countries with a gatekeeper and a GP-led primary care, i.e. Sweden and the Netherlands; (b) gatekeeper-mixed led: countries with a gatekeeper and either a paediatrician-led primary care, or a mixed paediatrician and GP-led primary care, i.e. Poland and Italy; (c) open access care: countries with care use without gatekeeping and lead practitioner, i.e. Germany and Cyprus.^4^^,^^20^ The countries with the three system types vary on a continuum with at the one end a centralized regulated professional model organized around primary care (type a), and at the other end a decentralized regulated model with primary care less at the core of the health system and allowing for more professional autonomy in providing services (type c).^23^

Second, we selected experts regarding the topics of the selected guidelines who also had a general view on child primary health care in the country concerned. The experts were recruited via members of the External Advisory Board of the MOCHA project or country agents.^24^ The latter regarded a network of one agent per country who acted as informant for obtaining data from country sources. To ensure inclusion of a wide variety of professionals, we asked the agents and board to identify at least two experts in each country per field of the guidelines. The resulting number of approached experts per country varied: Italy 46, Germany 12, the Netherlands 9, Sweden 3, Cyprus 5 and Poland 19. These comprised varying backgrounds from care practice, policy making, expert and science centres, and from end users, i.e. patient or interest groups. All were selected because of their expertise on one or more of the selected topics of this study. All experts consented to the request of the country agent to participate in the questionnaire and were informed about the objectives of the questionnaire, details about the MOCHA research project, confidentiality of provided information by the experts and anonymous report of data by the researchers. According to the criteria of the Dutch Medical Research Involving Human Subjects Act, this study did not need to be submitted for ethical approval by a Medical Ethical Committee. The study was reviewed and approved by the ethical committee of the Faculty of Behavioural, Management and Social Sciences of the University of Twente under file number BCE17583, on 19 September 2017.

### Procedure and measures

Data were collected end of 2017 and beginning of 2018. The experts filled in an online questionnaire comprising three sections on child vaccination, mental health problems or asthma care. The measures used for these three topics were similar, but the respondents could choose for which one of these health topics they gave their answers and opinions. Participation was on a voluntary and anonymous basis. We used the following measures.

#### Availability

We asked about availability of the guidelines and procedures for each topic, i.e.: (i) under-vaccination of children, (ii) asthma care in children aged over 6 years, and (iii) identification or assessment of mental health problems in adolescents aged 10–18 years (see table 1).

**Table 1:** Questionnaire items to measure guideline availability, use, achieved delivery and implementation

Constructs	Questionnaire item
Guideline availability, use and delivery	
Availability	In your country, is a guideline or formal procedure formulated for [best practicea]^a^ Yes, at an international level, yes, at national level, yes, at regional level, yes, at international and national level, yes, at international and regional level, yes, at national and regional level, yes, at international, national and regional level, no
Use	In your country, how often do primary care practitioners use a guideline or formal procedure for [best practice]? Often, the guideline or formal procedure is used with nearly all children who have a significant likelihood of having [health theme].^b^ Sometimes, the guideline or formal procedure is used with a number of children who have a significant likelihood of having [health theme]. Hardly, such guideline or formal procedure is not used at all. I do not know.
Achieved delivery	In your opinion, to what extent do primary care practitioners implement the actions of the guideline or formal procedure^c^ in the intended way? To a great extent, somewhat, very little, not at all.
Implementation barriers and facilitators	
	The statements below relate [action of guideline or formal procedure] by primary care practitioners. If [action of guideline or formal procedure] is not performed by primary care practitioners in your country, then please also indicate what your opinion is. If you have comments, please feel free to write them down in the open space next to the answer question
Characteristics of the guidelines	
Procedural clarity	The guideline or formal procedure in my country clearly describes the subsequent actions to be taken by primary care practitioners for [action of guideline or formal procedure]. Strongly disagree (1) to strongly agree (5)
Correctness	The inclusion of [action of guideline or formal procedure] in the guideline or formal procedure in my country is based on factual correct knowledge. Strongly disagree (1) to strongly agree (5)
Complexity	The [action of guideline or formal procedure] is too complex to perform by [primary care doctors or practice nurses] in my country. Strongly disagree (1) to strongly agree (5)
Compatibility	The [action of guideline or formal procedure] fits well within the routine practice of primary care practitioners in my country. Strongly disagree (1) to strongly agree (5)
Characteristics of the primary care practitioner	
Outcome expectations	Primary care practitioners in my country think it is important to use [action of guideline or formal procedure]. Strongly disagree (1) to strongly agree (5) Primary care practitioners in my country expect that [action of guideline or formal procedure] will lead to identification of [health theme]. Strongly disagree (1) to strongly agree (5)
Professional obligation	Primary care practitioners in my country feel it as their responsibility to [action of guideline or formal procedure]. Strongly disagree (1) to strongly agree (5)
Knowledge	[Primary care doctors or practice nurses] in my country have the knowledge to [action of guideline or formal procedure]. Strongly disagree (1) to strongly agree (5)
Descriptive norm	The [action of guideline or formal procedure] is generally accepted by primary care practitioners in my country. Strongly disagree (1) to strongly agree (5)
Self-efficacy	[Primary care doctors or practice nurses] in my country have the skills to [action of guideline or formal procedure]. Strongly disagree (1) to strongly agree (5)
Organizational context	
Financial resources	There are enough financial resources available in my country for primary care practitioners to [action of guideline or formal procedure]*.* Strongly disagree (1) to strongly agree (5)
Time available	[Primary care doctors or practice nurses] in my country have sufficient time to [action of guideline or formal procedure] as intended in their routine practice. Strongly disagree (1) to strongly agree (5)
Material resources and facilities	Primary care practitioners have access to materials and other resources or facilities necessary to [action of guideline or formal procedure] as intended*.* Strongly disagree (1) to strongly agree (5)
Socio-political context	
Legislation and regulations	The [action of guideline or formal procedure] fits in well within the legislation and regulations in my country. Strongly disagree (1) to strongly agree (5)
Policy support	Health care policy makers in my country support [action of guideline or formal procedure]. Strongly disagree (1) to strongly agree (5)

a Guidelines address child vaccination of children, identification or assessment of mental health problems in adolescents aged 10–18 years, and diagnosis of asthma in children aged over 6 years.

b Health themes: parents who do not have their child vaccinated, adolescents with mental health problems, and children with asthma.

c Actions of guidelines: communication with parents who are not inclined to vaccinate their child, conduct of a risk assessment for mental health problems in adolescents aged 10–18 years, and performance of spirometry for diagnosing asthma in children aged over 6 years.

#### Use and achieved delivery

We asked how often primary care practitioners used the guidelines, ranging from use for all children with a significant likelihood of having the health problem concerned, to for hardly any children. This can also be understood as asking about coverage of the guideline, i.e. whether all the people who should be receiving the benefits of the guideline actually did so.^14^ Next, we assessed the achieved delivery of the guidelines.^14^ We did so by limiting questions to specific guideline actions professionals are commonly expected to perform. Therefore, we asked the extent to which the following guideline actions were implemented in the intended way: (i) communication with parents who are not inclined to vaccinate their child,^25^ (ii) conduct of a risk assessment for mental health problems in adolescents aged 10–18 years,^26^ and (iii) performance of spirometry for diagnosing asthma in children aged over 6 years^6–9^ (see table 1).

#### Barriers and facilitators

A total of sixteen statements on barriers for and facilitators of implementation of the guidelines were asked per specific action mentioned above, i.e. communication with parents about having their child vaccinated, risk assessment for mental health problems, and spirometry (table 1). The items originate from the Measurement Instrument for Determinants of Innovation (MIDI) and were grouped into four categories of facilitating or hindering factors,^18^ i.e. characteristics of the guidelines, the primary care practitioner, the organizational context, and the socio-political context.

#### Background characteristics

The questionnaire included questions on the type of organization where the experts were employed, their current position, their highest level of education, and their field of expertise.

### Analysis

First, we described the characteristics of the sample of experts participating in the study per country. Second, we assessed the availability of guidelines for each health topic in six EU countries using crosstabs, with country as independent variable. Then, we assessed the use and achieved delivery per guideline using crosstabs, with country as independent variable. Questions on implementation barriers and facilitators with Likert-type scales ranging from 1 to 5 were pooled reverse when stated negative. Per item and per respondent, answer categories strongly agree and agree were scored as positive (+), neither agree or disagree as neutral (0), and strongly disagree and disagree as negative (−). Per country these scores were compiled into schemes with items and categories. Next, at least two raters (P.L.K., N.M.C.v.K. and R.v.Z.) made total scores per category classified as positive (facilitator) or negative (barrier), as shown in Supplementary table S1. The SPSS package for statistical analysis was used.^27^

## Results

### Characteristics of the sample

We invited a total of 94 experts, of which 46 participated (response 49%, varying from 35% in Italy to 89% in the Netherlands). The respondents identified themselves predominantly as experts from practice or science (table 2). They considered themselves most knowledgeable on child vaccination and asthma care. The respondents’ most common affiliations were research institutes and universities or community types of primary care organizations. The respondents were active as paediatricians or nurses in paediatrics (in total 14), child and adolescent psychiatrists or psychologists (12), medical doctors in leading positions (11), among them two GPs, researchers (4) or other professions (5).

**Table 2:** Characteristics of study participants

	Sweden (*N* = 5)	Netherlands (*N* = 8)	Poland (*N* = 10)	Italy (*N* = 16)	Germany (*N* = 4)	Cyprus (*N* = 3)
Expertise best practice						
Child vaccination	2	5	3	6	2	2
Mental health	2	5	7	3	2	2
Asthma	2	5	2	9	2	1
Field of expertise						
Policy	−	−	−	2^a^	−	1
Practice	1	5	6	5	2	2
Knowledge and science	4	2	3	8	2	−
Patient or interest group	−	1	1	−	−	−
Type of organization						
Hospital	1	−	1	1	2	1
Research institute/university	2	2	2	7	2	−
Expert centre	1	1	−	−	−	1
Other	1^b^	5^c^	7^d^	8^e^		1^f^
Education						
Associate degree	−	−	−	3	−	−
Master’s degree	−	1	4	2		1
Professional degree	−	3	1	2	−	1
Doctorate degree	5	3	3	2	4	1
Other	−	1	2	6	−	−

a One respondent unknown.

b Government agency (*n* = 1).

c (Local) public health/primary care organizations (*n* = 5).

d Mental health services (*n* = 3), NGO (*n* = 1), outpatient centre (*n* = 2), unknown (*n* = 1).

e Primary care/family pediatrics/community based (*n* = 5), patient organization (*n* = 1), local health care company (*n* = 1), Ministry (*n* = 1).

f Private organization (*n* = 1).

### Availability, use and achieved delivery of guidelines

For three countries the experts agreed about the availability of guidelines for vaccination, for all six countries about guidelines for asthma care, and for three countries about those for mental health assessment (see table 3). An overview of the guidelines mentioned by the experts per topic and per country is given in Supplementary table S2. Guidelines for child vaccination were used for nearly all children and achieved delivery was as intended in most countries as perceived by the respondents. Countries differed largely regarding use and achieved delivery of mental health assessment guidelines. Guidelines for asthma care were available according to most experts, but they were only used for all children at risk in Sweden and Poland. Implementation as intended of the asthma guideline, i.e. performance of spirometry, was poor in all countries, except Sweden.

**Table 3:** Availability, use and achieved delivery of guidelines per country

	Availability of guidelines^a^			Use of guidelines^b^			Achieved delivery^c^		
	Child vaccination	Mental health	Asthma	Child vaccination	Mental health	Asthma	Child vaccination	Mental health	Asthma
Sweden	+	+/−	+	−	^d^	+	−	+	+
Netherlands	+/−	+	+	+	+	−	+	−	−
Poland	+	+	+	+	−	+	+	+/−	−
Italy	+	+/−	+	+	−	−	+	+	−
Germany	+/−	+	+	+	+	−	+	+	−
Cyprus	+/−	−	+	+	^d^	−	−	^d^	−

a + available, − not available, ± answers varied.

b + use for nearly all children, − sometimes or hardly for any children.

^c^ + to a great or certain extent implemented as intended, − somewhat or very little implemented as intended, +/- answers varied.

d Missing value.

### Facilitators and barriers

Important facilitators in communicating with vaccination hesitant parents were the characteristics of the guideline, e.g. not too difficult to perform and fitting well within routine practice (table 4 and Supplementary table S2). Also, the general acceptance by primary care practitioners and the socio-political context of support for child vaccination from health care policy makers were mentioned as facilitators. Financial constraints and limited time available were mentioned as important barriers for use of vaccination guidelines. All country experts except those from Poland mentioned mainly facilitators for all implementation categories regarding this activity.

**Table 4:** Overall scores for facilitators of and for barriers of implementation of guidelines per country

	Sweden	Netherlands	Poland	Italy	Germany	Cyprus
Communication to vaccinate child						
Characteristics guideline	+	+	0	0	+	+
Characteristics practitioner	+	+	−	+	+	+
Organizational context	−	−	−	0	+	+
Socio-political context	+	+	−	+	+	0
Risk assessment mental health						
Characteristics guideline	−	+	0	0	+	−
Characteristics practitioner	−	+	0	0	+	0
Organizational context	−	0	−	0	−	−
Socio-political context	0	+	0	0	−	−
Spirometry						
Characteristics guideline	+	0	+	0	0	0
Characteristics practitioner	+	−	+	−	0	0
Organizational context	0	−	−	−	0	+
Socio-political context	+	+	0	+	0	−

+, facilitator; −, barrier; 0, facilitator and barrier.

For conducting a risk assessment of mental health problems in adolescents the experts brought forward the barriers lack of knowledge or skills of professionals, lack of time and funds, and lack of dedicated policies. Facilitators were training of professionals, options for referral, financial resources and legislation. Experts from the Netherlands mentioned almost only facilitators, whereas experts from Sweden and Cyprus mentioned mainly barriers, and experts from Germany, Italy and Poland both barriers and facilitators.

Concerning implementation of the asthma guideline, in particular spirometry, experts from almost all countries identified barriers related to practitioners’ characteristics such as lack of knowledge and self-efficacy of doctors and nurses, and at organizational level such as limited financial resources and time available. Moreover, barriers existed at the level of the socio-political context, namely lack of fit with legislation and regulations and lack of policy support. Facilitators regarded a foundation in correct knowledge as characteristic of the guideline and its fit with legislation and regulations.

Availability, use and achieved delivery of guidelines regarding the three selected topics seemed to be fairly independent from the type of primary health care system. The experts from the Netherlands and Sweden, countries with gatekeeper GP-led systems, experienced facilitators for guideline implementation to a slightly greater extent and some less barriers, in the Netherlands particularly in risk assessment of adolescent mental health problems and in Sweden in use of spirometry.

## Discussion

We conducted a qualitative case study on the availability of clinical guidelines, their use and achieved delivery by child primary health care professionals, and the facilitators and barriers of implementation of the guidelines in six European countries. According to most countries’ experts, guidelines were generally available. Use of guidelines and achieved delivery as intended were favourable for child vaccination but relatively poor for mental health assessment and asthma care. Factors affecting the implementation of the guidelines differed, but barriers were notably found regarding mental health assessment and asthma care, i.e. risk assessment for mental health problems and performing spirometry. We found no clear relationship of use and achieved delivery of the guidelines with type of child primary health care system.

We found ample availability of guidelines for primary child health care which may be explained by strong governance, e.g. either state governance in centrally led countries such as observed in Sweden or the Netherlands, or strong clinical governance manifest in professional and organizational accountability as observed in less centrally led countries, such as Germany.^28^ Another explanation for guideline availability may regard a country’s financial resources and associated expenditures on health care. This could explain the advantaged position of Germany in guideline availability in comparison to Cyprus with a much lower Gross Domestic Product and lower health expenditure per capita than Germany, which both regard countries with open access care. Both governance and financial resources may thus be important factors for the availability of guidelines for child health care.

We found use and delivery of the guidelines mental health assessment and for asthma care to be much poorer than for child vaccination. A first explanation for this difference may regard the characteristics of the guideline. For child vaccination the good fit of the guidelines with daily routines added to the uptake in most countries according to the experts, whereas the complexity of carrying out the guideline for asthma care^29^ may explain its poor delivery. This wide variation in guideline uptake per targeted health problem confirms previous findings, with uptake ranging from 28% for a guideline regarding sudden infant death to 100% for a guideline on congenital heart disorders.^11^ These findings show that use and delivery of a guideline depends on the type of health problem addressed combined with characteristics of the guideline such as its complexity.

A second explanation for the differences in use and delivery of guidelines as found may regard the characteristics of practitioners in a specific country, like their attitudes, knowledge and skills. The general acceptance of the child vaccination guidelines explains its favourable implementation. The practitioners’ knowledge and skills seemed to be important for the use and delivery of the high uptake of the asthma care guideline in Sweden. Research has shown the presence of specialized asthma nurses in primary health care clinics to add to performing spirometry tests in Sweden.^29^ The large investment in obtaining evidence for best psychosocial assessment by primary health care physicians and nurses in the Netherlands, resulting in a broadly used guideline since 2000, is another example of the practitioners’ role in guideline implementation.^30^^,^^31^ However, the Dutch experts were still critical about the delivery as intended of that guideline. Our study here shows the importance of practitioners’ characteristics attitude, knowledge and skills for use and delivery of guidelines. Finally, our findings show that professionals differ in what they consider to be suitable as guideline, e.g. Dutch and German experts varied regarding the reported availability of vaccination guidelines. Probably this variation may be understood as that some experts consider a child vaccination scheme with some additional information as a sufficient guideline whereas others do not.

We found that factors in the organizational and socio-political context facilitated or hampered guideline implementation, but that the occurrence of such factors had no clear relationship with type of primary child health care system in these six countries. The time and cost that experts mentioned as organizational barriers have been reported before^32^^,^^33^ and the same holds for the importance of adequate training of professionals for guideline adherence.^32^^,^^34^^,^^35^ Regarding the socio-political context, experts frequently mentioned lack of policy support as barrier for guideline implementation. However, this did not affect adherence to guidelines for child vaccination as these seem generally accepted by professionals and also the social-political context seems favourable in most countries. Such policy support for planned and structured guideline implementation will have to take into account the countries’ epidemiological, socio-cultural, socio-economic, ethical, legal, and political circumstances.^36–38^ Organizational context and policy support should thus be addressed in the implementation of guidelines for child primary health care.

### Strengths and limitations

A strength of this study is its case study approach in which we compared guidelines regarding three selected topics covering a broad range of age categories of children and adolescents, a range of service functions, i.e. prevention, surveillance and diagnosis in primary care, including somatic and mental health care, and a range of contexts and health care systems among EU countries, i.e. centralized or decentralized regulated models. In this way we got a fair representative picture of topics and health care systems, relevant for qualitative assessment of guideline use and implementation. A second strength is our use of a validated scale measuring implementation in child primary health care,^18^ combined with qualitative data on the availability and use of guidelines.

A limitation is that experts mainly came from practice, expert centres, and science with only two representing patient organizations. We may thus not have gained full insight in the achieved delivery of guidelines from the perspective of patients. A second limitation is the small number of participating experts from Sweden, Germany and Cyprus relative to the numbers from the other countries. Our findings thus require confirmation for these relatively underrepresented countries. Moreover, we mostly obtained expert opinions from the field of paediatrics and relatively few from general practice. Future studies should also address more experts from the latter field.

### Implications for practice

This study showed room for improvement of guideline availability, use and achieved delivery with variation in the health problem addressed and practitioners’ characteristics. Education on the awareness of the usefulness of guideline recommendations and systematic postgraduate training of skills are advised to support the adherence to guidelines in routine daily practice, often tasks of great complexity.^29^ Training of professionals enables them to use the guideline for the patient ‘tailor-made’ and provides the practitioner with substantiated arguments to use the guideline. Training of professionals, time and financial resources as part of the organizational context of primary child health care should facilitate guideline implementation.

Furthermore, our research shows a clear demand for policy support for structured implementation of guidelines for adolescents’ mental health assessment and asthma care. Our findings suggest that strong governance, i.e. state regulation or clinical leadership, and sufficient health care expenditures are associated with better guideline availability and use. This may evidently lead to major gains in child and youth health. We therefore strongly recommend support from national governments and paediatric and public health associations for guidelines, embedded in a strong quality assurance system. Clear policy making and increase of resources could benefit such quality systems.^39^

### Implications for research

Availability, use and achieved delivery of guidelines varied largely, with variation only partially explained, showing a need for further research to better understand the way systems of child primary care influence the implementation of guidelines. First, this requires structured monitoring and evaluation of guideline implementation using standardized measurement instruments, such as the MIDI or the GuideLine Implementability Appraisal (GLIA) instrument.^18^^,^^40^ Second, we need further research to increase our understanding of the effects of external factors on perceived facilitators and barriers of implementation, such as the influence of the availability of guidelines and degree of experience with guidelines in practice.

## Conclusions

This study shows varying degrees of availability, use and achieved delivery of clinical guidelines in child primary health care in six EU countries. Adequate training of professionals, compatibility with daily routines, strong governance and sufficient financial resources seemed important facilitators for guideline implementation, though implementation conditions in the countries can be strongly improved. This can yield major gains in child and youth health across Europe.

## Supplementary data

Supplementary data are available at *EURPUB* online.

## Supplementary Material

ckac114_Supplementary_Data

## Data Availability

The data underlying this article are available in DANS (Data Archiving and Networked Service) repository at DOI: https://doi.org/10.17026/dans-28v-cwcf.

## References

[1] World Health Organization, European Union for School and University Medicine, editors. *European Framework for Quality Standards in School Health Services and Competences for School Health Professionals [Internet]*. Copenhagen: WHO Regional Office for Europe, 2014. Available at: https://www.euro.who.int/__data/assets/pdf_file/0003/246981/European-framework-for-quality-standards-in-school-health-services-and-competences-for-school-health-professionals.pdf (6 January 2022, date last accessed).

[2] World Health Organization. *Making Health Services Adolescent Friendly: Developing National Quality Standards for Adolescent-Friendly Health Services [Internet].* Geneva: World Health Organization, 2012. Available at: https://apps.who.int/iris/bitstream/handle/10665/75217/9789241503594_eng.pdf (6 January 2022, date last accessed).

[3] World Health Organization, United Nations Children’s Fund, World Bank Group. *Nurturing Care for Early Childhood Development: A Framework for Helping Children Survive and Thrive to Transform Health and Human Potential [Internet].* Geneva: World Health Organization, 2018. Available at: https://apps.who.int/iris/bitstream/handle/10665/272603/9789241514064-eng.pdf (6 January 2022, date last accessed).

[4] Blair M , Rigby M, Alexander D, editors. Issues and Opportunities in Primary Health Care for Children in Europe: The Final Summarised Results of the Models of Child Health Appraised (MOCHA) Project [Internet]. Bingley: Emerald Publishing, 2019. Available at: 10.1108/9781789733518 (6 January 2022, date last accessed).

[5] Woolf SH , Grol R, Hutchinson A, et al Clinical guidelines: potential benefits, limitations, and harms of clinical guidelines. BMJ 1999;318:527–30. 10024268 10.1136/bmj.318.7182.527 PMC1114973

[6] Global Initiative for Asthma. Global Strategy for Asthma Management and Prevention [Internet]. Global Initiative for Asthma, 2016. Available at: https://ginasthma.org (30 April 2021, date last accessed).

[7] Reddel HK , FitzGerald JM, Bateman ED, et al GINA 2019: a fundamental change in asthma management: treatment of asthma with short-acting bronchodilators alone is no longer recommended for adults and adolescents: treatment of asthma with short-acting bronchodilators alone is no longer recommended for adults and adolescents. Eur Respir J 2019;53:1901046. 31249014 10.1183/13993003.01046-2019

[8] Gaillard EA , Kuehni CE, Turner S, et al European Respiratory Society clinical practice guidelines for the diagnosis of asthma in children aged 5–16 years. Eur Respir J 2021;58:2004173. 33863747 10.1183/13993003.04173-2020

[9] Gaillard EA , Moeller A. Evidence-based European guidelines for the diagnosis of asthma in children aged 5–16 years. Lancet Respir Med 2021;9:558–60. 33894141 10.1016/S2213-2600(21)00183-1

[10] Durlak JA , DuPre EP. Implementation matters: a review of research on the influence of implementation on program outcomes and the factors affecting implementation. Am J Community Psychol 2008;41:327–50. 18322790 10.1007/s10464-008-9165-0

[11] Fleuren MAH , van Dommelen P, Dunnink T. A systematic approach to implementing and evaluating clinical guidelines: the results of fifteen years of preventive child health care guidelines in the Netherlands. Soc Sci Med 2015;136–137:35–43. 10.1016/j.socscimed.2015.05.001 25982867

[12] Cook DA , Pencille LJ, Dupras DM, et al Practice variation and practice guidelines: attitudes of generalist and specialist physicians, nurse practitioners, and physician assistants. PLoS One 2018;13:e0191943. 29385203 10.1371/journal.pone.0191943 PMC5792011

[13] Kovacs E , Strobl R, Phillips A, et al Systematic review and meta-analysis of the effectiveness of implementation strategies for non-communicable disease guidelines in primary health care. J Gen Intern Med 2018;33:1142–54. 29728892 10.1007/s11606-018-4435-5 PMC6025666

[14] Carroll C , Patterson M, Wood S, et al A conceptual framework for implementation fidelity. Implement Sci 2007;2:40. 18053122 10.1186/1748-5908-2-40 PMC2213686

[15] Boluyt N , Lincke CR, Offringa M. Quality of evidence-based pediatric guidelines. Pediatrics 2005;115:1378–91. 15867050 10.1542/peds.2004-0575

[16] Liu Y , Zhang Y, Wang S, et al Quality of pediatric clinical practice guidelines. BMC Pediatr 2021;21:223–31. 33962599 10.1186/s12887-021-02693-1 PMC8103635

[17] Wilby KJ , Black EK, MacLeod C, et al Critical appraisal of clinical practice guidelines in pediatric infectious diseases. Int J Clin Pharm 2015;37:799–807. 25910479 10.1007/s11096-015-0123-2

[18] Fleuren MAH , Paulussen TGWM, Van Dommelen P, Van Buuren S. Towards a measurement instrument for determinants of innovations. Int J Qual Health Care 2014;26:501–10. 24951511 10.1093/intqhc/mzu060 PMC4195468

[19] Grol R , Dalhuijsen J, Thomas S, et al Attributes of clinical guidelines that influence use of guidelines in general practice: observational study. BMJ 1998;317:858–61. 9748183 10.1136/bmj.317.7162.858 PMC31096

[20] van Esso D , del Torso S, Hadjipanayis A, et al; Primary-Secondary Working Group (PSWG) of European Academy of Paediatrics (EAP). Paediatric primary care in Europe: variation between countries. Arch Dis Child 2010;95:791–5. 20403821 10.1136/adc.2009.178459

[21] Gerring J. The case study: what it is and what it does. In: Goodin RE, editor. The Oxford Handbook of Political Science. Oxford: Oxford University Press, 2011.

[22] Yin RK. Case study research: design and methods. Thousand Oaks, California: SAGE Publications, 2002.

[23] Bourgueil Y , Marek A, Mousquès J. Three models of primary care organization in Europe, Canada, Australia and New-Zealand. Paris: Irdes, Issues in Health Economics 2009;1–6. Available at https://www.irdes.fr/EspaceAnglais/Publications/IrdesPublications/QES141.pdf(22 August 2022, date last accessed).

[24] Blair M , Alexander D, Rigby M. The MOCHA project: origins, approach and methods. In: Blair M, Rigby M, Alexander D, editors. Issues and Opportunities in Primary Health Care for Children in Europe: The Final Summarised Results of the Models of Child Health Appraised (MOCHA) Project [Internet]. Bingley: Emerald Publishing, 2019. Available at: 10.1108/9781789733518.

[25] Rainey JJ , Watkins M, Ryman TK, et al Reasons related to non-vaccination and under-vaccination of children in low and middle income countries: findings from a systematic review of the published literature, 1999-2009. Vaccine 2011;29:8215–21. 21893149 10.1016/j.vaccine.2011.08.096

[26] State of Western Australia. Working With Youth—A Legal Resource for Community-Based Health Professionals. Perth: WA Country Health Service, 2020.

[27] IBM Corp. IBM SPSS Statistics for Windows, Version 25.0. Armonk, New York: IBM Corp, 2017.

[28] Spyridonidis D , Calnan M. Implementing clinical governance policy: NICE. Br J Health Care Manag 2010;16:394–401.

[29] Jonsson M , Egmar A-C, Kiessling A, et al Adherence to national guidelines for children with asthma at primary health centres in Sweden: potential for improvement. Prim Care Respir J 2012;21:276–82. 22751738 10.4104/pcrj.2012.00051 PMC6547951

[30] Brugman E , Reijneveld SA, Verhulst FC, Verloove-Vanhorick SP. Identification and management of psychosocial problems by preventive child health care. Arch Pediatr Adolesc Med 2001;155:462–9. 11296073 10.1001/archpedi.155.4.462

[31] Reijneveld SA , de Meer G, Wiefferink CH, Crone MR. Parents’ concerns about children are highly prevalent but often not confirmed by child doctors and nurses. BMC Public Health 2008;8:124. 18423036 10.1186/1471-2458-8-124 PMC2383909

[32] O’Brien D , Harvey K, Howse J, et al Barriers to managing child and adolescent mental health problems: a systematic review of primary care practitioners’ perceptions. Br J Gen Pract 2016;66:e693–707. 27621291 10.3399/bjgp16X687061 PMC5033306

[33] Siciliani L , Wild C, McKee M, et al Strengthening vaccination programmes and health systems in the European Union: a framework for action. Health Policy 2020;124:511–8. 32276852 10.1016/j.healthpol.2020.02.015

[34] Kaminsky DA , Marcy TW, Bachand M, Irvin CG. Knowledge and use of office spirometry for the detection of chronic obstructive pulmonary disease by primary care physicians. Respir Care 2005;50:1639–48. 16318645

[35] Goeman DP , Hogan CD, Aroni RA, et al Barriers to delivering asthma care: a qualitative study of general practitioners. Med J Aust 2005;183:457–60. 16274345 10.5694/j.1326-5377.2005.tb07122.x

[36] Schloemer T , Schröder-Bäck P. Criteria for evaluating transferability of health interventions: a systematic review and thematic synthesis. Implement Sci 2018;13:88. 29941011 10.1186/s13012-018-0751-8 PMC6019740

[37] Panteli D , Legido-Quigley H, Reichebner C, et al Clinical practice guidelines as a quality strategy. In: Busse R, Klazinga N, Panteli D, Quentin W, editors. Improving Healthcare Quality in Europe Characteristics, Effectiveness and Implementation of Different Strategies. Copenhagen: WHO Regional Office for Europe, 2019: 233–64. 31721544

[38] Fischer F , Lange K, Klose K, et al Barriers and strategies in guideline implementation—a scoping review. Healthcare (Basel) 2016;4:36. 10.3390/healthcare4030036 PMC5041037 27417624

[39] Burgers JS , Cluzeau FA, Hanna SE, et al Characteristics of high-quality guidelines: evaluation of 86 clinical guidelines developed in ten European countries and Canada. Int J Technol Assess Health Care 2003;19:148–57. 12701947 10.1017/s026646230300014x

[40] Shiffman RN , Dixon J, Brandt C, et al The GuideLine Implementability Appraisal (GLIA): development of an instrument to identify obstacles to guideline implementation. BMC Med Inform Decis Mak 2005;5:23. 16048653 10.1186/1472-6947-5-23 PMC1190181

