# Supplementary material for: Availability and implementation of guidelines in European child primary health care: how can we improve?
Source: Eur J Public Health. 2022 Aug 25;32(5):670–6. doi: 10.1093/eurpub/ckac114 (PMC9527973; doi:10.1093/eurpub/ckac114)
Supplement: ckac114_Supplementary_Data [file ckac114_supplementary_data.docx]

Supplementary Table 1. Experts’ answers on MIDI questionnaire items about guideline implementation facilitators and barriers per country, as summarized in table 4 of the paper

|  | Sweden | Netherlands | Poland | Italy | Germany | Cyprus |
| --- | --- | --- | --- | --- | --- | --- |
| ***Communication to vaccinate child*** | | | | | | |
| **Characteristics guideline** -procedural clarity - correctness - complexity   *doctor  nurse* - compatibility | NA NA  + (n=2)^$^ + (n=2)  + (n=1) / - (n=1) | NA NA  + (n=4) / 0 (n=1 ) + (n=3), 0 (n=2 ) 0 (n=1), + (n=4) | NA NA  + (n=1) / - (n=2) + (n=1) / - (n=2) - (n=1) / + (n=2) | NA NA  + (n=5) / (n=1) + (n=2) / 0 (n=1) / - (n=3)  - (n=2) / 0 (n=1) / + (n=3) | NA NA  + (n=2) 0 (n=1) - (n=1) / + (n=1) | NA NA  + (n=2) - (n=1) / + (n=1) + (n=2) |
| Total score | + | + | 0 | 0 | + | + |
| **Characteristics practitioner -** outcome expectations  *importance*  *objectives reached*  - professional obligation - knowledge  *doctor*  *nurse* - self-efficacy  *doctor*  *nurse -* descriptive norm | + (n=1) + (n=1) + (n=1)  + (n=1) + (n=2)  0 (n=1) 0 (n=1) / + (n=1) + (n=1) | - (n=1) / + (n=4) - (n=1) / 0 (n=1) / + (n=3) - (n=1) / + (n=4)  - (n=1) / + (n=4) - (n=1) / 0 (n=1) / + (n=3)  - (n=1) / + (n=4) - (n=1) / 0 (n=1) / + (n=3) - (n=1) / + (n=4) | + (n=2) + (n=2) + (n=2)  - (n=1) / 0 (n=1) - (n=1) / 0 (n=1)  - (n=1) / 0 (n=1) - (n=1) / 0 (n=1)  - (n=1) / + (n=1) | - (n=1) / + (n=5) 0 (n=2) / + (n=4) - (n=1) / 0 (n=1) / + (n=4)  - (n=1) / 0 (n=3) / + (n=2) - (n=4) / 0 (n=1) / + (n=1)  - (n=1) / 0 (n=3) / + (n=2) - (n=4) / 0 (n=1) / + (n=1) - (n=1) / + (n=5) | + (n=1) 0 (n=1) + (n=1)  + (n=1) 0 (n=1)  + (n=1) 0 (n=1) + (n=1) | 0 (n=1 / + (n=1) 0 (n=1 / + (n=1) + (n=2)  - (n=1) / + (n=1) - (n=1) / + (n=1)  - (n=1) / + (n=1) - (n=1) / + (n=1) 0 (n=1) / + (n=1) |
| Total score | + | + | - | + | + | + |
| **Organizational context -** financial resources - time available  *doctor*  *nurse* - material resources and facilities | - (n=1)  - (n=2) - (n=2) + (n=2) | - (n=4) / + (n=1)  - (n=4) / + (n=1) - (n=4) / + (n=1) 0 (n=2) / + (n=3) | - (n=2) / 0 (n=1)  - (n=2) / 0 (n=1) - (n=1) / 0 (n=1) / + (n=1) - (n=2) / 0 (n=1) | - (n=3) / + (n=3)  - (n=3) / + (n=3) - (n=3) / 0 (n=2) / + (n=1) - (n=2) / + (n=4) | + (n=1)  0 (n=1) 0 (n=1) + (n=1) | + (n=2)  + (n=2)  + (n=2) 0 (n=2) |
| Total score | - | - | - | 0 | + | + |
| **Socio-political context**  - legislation and regulations - policy support | + (n=2)  + (n=2) | - (n=2) / + (n=3) - (n=1) / + (n=4) | - (n=1) / 0 (n=2) - (n=1) / 0 (n=2) | 0 (n=2) / + (n=4) - (n=3) / + (n=3) | + (n=1) + (n=1) | - (n=1) / 0 (n=1) 0 (n=1) / + (n=1) |
| Total score | + | + | - | + | + | 0 |
|  | **Sweden** | **Netherlands** | **Poland** | **Italy** | **Germany** | **Cyprus** |
| ***Risk assessment mental health*** | | | | | | |
| **Characteristics guideline** -procedural clarity - correctness - complexity   *doctor  nurse* - compatibility | * * * - (n=1)^$^ - (n=1) - (n=1) | 0 (n=1), + (n=2) - (n=1) / + (n=2)  + (n=4) + (n=3) / 0 (n=1) + (n=4) | - (n=2) / + (n=1) - (n=1) / 0 (n=1 / + (n=1)  + (n=3) / 0 (n=2) / - (n=1) + (n=1) / 0 (n=1) / - (n=4) + (n=2) / 0 (n=1) / - (n=3) | -(n=1) / + (n=1) 0 (n=1) / + (n=1)  + (n=2) / - (n=1) + (n=2) / - (n=-1) - (n=1) / 0 (n=1) / + (n=1) | - (n=1) / + (n=1) + (n=1)  + (n=2) + (n=2) 0 (n=1) / + (n=1) | * *  - (n=1) - (n=1) 0 (n=1) |
| Total score | - | + | 0 | 0 | + | - |
| **Characteristics practitioner**  - outcome expectations  *importance*  *objectives reached* - professional obligation - knowledge  *doctor*  *nurse* - self-efficacy  *doctor*  *nurse*  *-* descriptive norm | - (n=1) - (n=1) - (n=1)  - (n=1) - (n=1)  - (n=1) - (n=1) 0 (n=1) | + (n=4) + (n=4) + (n=4)  0 (n=2) / + (n=2) 0 (n=2) / + (n=2)  - (n=1) / + (n=3) 0 (n=3) / + (n=1) 0 (n=1) / + (n=3) | - (n=1) / 0 (n=1) / + (n=3) - (n=2) / 0 (n=1) / + (n=2) - (n=2) / 0 (n=1) / + (n=2)  - (n=3), 0 (n=1) / + (n=1)  - (n=4) / 0 (n=1)  - (n=3), 0 (n=1), + (n=1)  - (n=4), 0 (n=1)  - (n=1) / 0 (n=1) + (n=3) | 0 (n=1) / + (n=2) + (n=3) 0 (n=2) / + (n=1)  - (n=3) - (n=2) / 0 (n=1)  - (n=2) / + (n=1) - (n=2) / + (n=1) - (n=2) / 0 (n=1) | + (n=2) + (n=2) + (n=2)  0 (n=2) 0 (n=1)  - (n=1) / 0 (n=1) - (n=1) 0 (n=2) | 0 (n=1) 0 (n=1) 0 (n=1)  0 (n=1) - (n=1)  0 (n=1) - (n=1) 0 (n=1) |
| Total score | - | + | 0 | 0 | + | 0 |
| **Organizational context -** financial resources - time available  *doctor*  *nurse* - material resources and facilities | - (n=1)  - (n=1) - (n=1) - (n=1) | - (n=2) / 0 (n=1) / + (n=1)  - (n=2) / 0 (n=1) / + (n=1) - (n=1) / 0 (n=2) / + (n=1) - (n=1) / 0 (n=1) / + (n=2) | - (n=3), + (n=2)  - (n=4), 0 (n=1) - (n=4), 0 (n=1) - (n=4) / 0 (n=1) | - (n=2) / + (n=1)  - (n=2) / + (n=1) - (n=2) / + (n=1) - (n=1) / + (n=2) | - (n=1) / 0 (n=1)  - (n=2) - (n=1) - (n=2) | 0 (n=1)  0 (n=1) - (n=1) - (n=1) |
| Total score | - | 0 | - | 0 | - | - |
| **Socio-political context**  - legislation and regulations - policy support | + (n=1) 0 (n=1) | + (n=4) 0 (n=1) / + (n=3) | - (n=1) / + (n=4) - (n=4) / + (n=1) | - (n=2) / + (n=1) - (n=1) / 0 (n=1) / + (n=1) | - (n=1) / 0 (n=1) - (n=2) | - (n=1) - (n=1) |
| Total score | 0 | + | 0 | 0 | - | - |
|  | **Sweden** | **Netherlands** | **Poland** | **Italy** | **Germany** | **Cyprus** |
| ***Spirometry*** |  |  |  |  |  |  |
| **Characteristics guideline** -procedural clarity - correctness - complexity   *doctor  nurse* - compatibility | + (n=1)^$^ + (n=1)  + (n=1) + (n=1) + (n=1) | - (n=2)/ 0 (n=2) / + (n=1) 0 (n=3) / + (N=2)  + (n=3) / 0 (n=2) + (n=2) / 0 (n=1) / - (n=2) - (n=2) / 0 (n=3) | - (n=1 ) / + (n=1) + (n=2)  + (n=1) / - (n=1) + (n=1) / - (n=1) + (n=2) | - (n=4) / 0 (n=1) / +( n=3) - (n=1) / 0 (n=1) / + (n=6)  + (n=3) / 0 (n=2) / - (n=3) + (n=4) / - (n=4) - (n=5) / 0 (n=1) / +(n=2) | - (n=1) / + (n=1) + (n=1)  - (n=1) / + (n=1) - (n=1) / + (n=1) - (n=1) / + (n-1) | 0 (n=1) + (n=1)  0 (n=1) 0 (n=1) 0 (n=1) |
| Total score | + | 0 | + | 0 | 0 | 0 |
| **Characteristics practitioner** - outcome expectations  *importance*  *objectives reached* - professional obligation - knowledge  *doctor*  *nurse* - self-efficacy  *doctor*  *nurse*  *-* descriptive norm | + (n=1) + (n=1) 0 (n=1)  + (n=1) + (n=1)  + (n=1) + (n=1) + (n=1) | - (n=3) / 0 (n=1) / + (n=1) - (n=3) / 0 (n=1) / + (n=1) - (n=4) / 0 (n=1)  - (n=3) / 0 (n=1) / + (n=1) - (n=2) / 0 (n=1) / + (n=2)  - (n=2) / 0 (n=3) - (n=1) / 0 (n=2) / + (n=2) - (n=3) / 0 (n=1) / + (n=1) | + (n=2) + (n=2) + (n=2)  + (n=2) + (n=1) / 0 (n=1)  + (n=1) / 0 (n=1) 0 (n=2) + (n=2) | - (n=3) / 0 (n=2) / + (n=2) - (n=2) / 0 (n=2) / + (n=3) - (n=5) / 0 (n=2)  - (n=3) / 0 (n=3) / + (n=1) - (n=5) / 0 (n=2)  - (n=6) / + (n=1) - (n=5) / + (n=2) - (n=4) / 0 (n=1) / + (n=2) | - (n=1) / + (n=1) - (n=1) / + (n=1) - (n=1) / + (n=1)  0 (n=1) / + (n=1) - (n=1) / + (n=1)  + (n=1) / - (n=1) + (n=1) / - (n=1) + (n=1) | + (n=1) + (n=1) + (n=1)  0 (n=1) - (n=1)  - (n=1) - (n=1) 0 (n=1) |
| Total score | + | - | + | - | 0 | 0 |
| **Organizational context -** financial resources - time available  *doctor*  *nurse*  - material resources and facilities | 0 (n=1)  - (n=1) + (n=1)  0 (n=1) | - (n=3) / 0 (n=1) / + (n=1)  - (n=3) / 0 (n=1) / + (n=1) 0 (n=3) / + (n=2)  - (n=2) / 0 (n=2) / + (n=1) | - (n=2)  - (n=2) + (n=1) / - (n=1)  + (n=2) | - - (n=5) / + (n=2)  - (n=6) / + (n=1) - (n=6 ) / + (n=1) - (n=2) / 0 (n=4 ) / + (n=1) | - (n=1) / + (n=1)  - (n=1) / 0 (n=1) - (n=1) / + (n=1)  - (n=1) / + (n=1) | +(n=1)  +(n=1) 0(n=1)  0 (n=1) |
| Total score | 0 | - | - | - | 0 | + |
| **Socio-political context**  - legislation and regulations - policy support | + (n=1) + (n=1) | 0 (n=1) / + (n=4) - (n=1) / 0 (n=2) / + (n=2) | + (n=2) - (n=1) / 0 (n=1) | 0 (n=1) / + (n=6) - (n=1) / + (n=6) | 0 (n=1) / + (n=1) - (n=1) / + (n=1) | - (n=1) 0 (n=1) |
| Total score | + | + | 0 | + | 0 | - |

+ facilitator
 - barrier
 0 facilitator and barrier
* missing

$ n=number of experts

Supplementary table 2. Guidelines or formal procedures used in child primary health care on the topics vaccination, mental health and asthma as mentioned by experts of six EU countries

|  | Guideline or formal procedure, author |
| --- | --- |
| Sweden | [www.rikshandboken-bhv.se](http://www.rikshandboken-bhv.se) , Rikshandboken for Professionen (National Handbook for the Profession)  Tailoring Immunization Programmes (TIP), WHO Europe |
|  | ***Child vaccination*** |
| Netherlands | <http://www.rivm.nl/Onderwerpen/R/Rijksvaccinatieprogramma> , Rijksinstituut voor Volksgezondheid en Milieu (RIVM, National Institute for Public Health and the Environment) |
| Poland | <https://gis.gov.pl/images/ep/so/pso_2017_-_nowelizacja.pdf> , Glówny Inspektorat Sanitary (Main Sanitary Inspectorate)  <http://prawo.sejm.gov.pl/isap.nsf/DocDetails.xsp?id=WDU20082341570> , Strona główna Sejmu Rzeczpospolitej Polskiej (Polish Health Care Law) |
| Italy | <https://www.epicentro.iss.it/en/> , Istituto Superiore di Sanità (Higher Institute of Health), EpiCentro  National vaccine prevention plan, Ministry of Health |
| Germany | <http://www.rki.de/DE/Content/Infekt/Impfen/impfen_node.html> , Robert Koch Institut (Robert Koch Institute) |
| Cyprus | - |
|  | ***Mental health*** |
| Sweden | <https://snpf.barnlakarforeningen.se/vardprogram-2/> , Svensk Neuropediatrisk Förening (SNPF, Swedish Neuropediatric Association)  <http://www.sfbup.se/vardprogram/> , Svenska föreningen för barn- och ungdomspsykiatri (SFBUP, Swedish Association for Child and Adolescent Psychiatry)  <https://lakemedelsverket.se/.../Depression-angestsyndrom-och-tvangssyndrom-hos-bar> , Läkemedelsverket, (Swedish Medical Products Agency)  [www.rikshandboken-bhv.se](http://www.rikshandboken-bhv.se) , Rikshandboken for Professionen (National Handbook for the Profession) |
| Netherlands | <https://www.nhg.org/actueel/dossiers/dossier-geestelijke-gezondheidszorg-ggz> , Nederlands Huisartsen Genootschap (NHG, Dutch College of General Practitioners)  <https://www.ncj.nl/richtlijnen/alle-richtlijnen/richtlijn/psychosociale-problemen> , Nederlands Centrum Jeugdgezondheid (NCJ, Netherlands Center Youth Health) |
| Poland | Polish Mental Health Care Law |
| Italy | <http://sinpia.eu/> , Societa Italiana di Neurpsychiatria dell’Infanzia e dell’Adolescenza (Italian Society of Childhood and Adolescent Neuropsychiatry) |
| Germany | - |
| Cyprus | - |
|  | ***Asthma*** |
| Sweden | <https://www.lakemedelsverket.se/sv> , Läkemedelsverket, (Swedish Medical Products Agency)  <http://slmf.se/wp-content/uploads/2018/08/vard-vid-astma-och-kol-2015.pdf> , Vård vid astma och KOL Stöd för styrning och ledning (National guideline Asthma care and COPD Support for control and management) |
| Netherlands | <https://www.ncj.nl/richtlijnen/alle-richtlijnen/richtlijn/astma-2020> , Nederlands Centrum Jeugdgezondheid (Netherlands Center Youth Health)  <https://richtlijnen.nhg.org/multidisciplinaire-richtlijnen/astma-bij-kinderen> , Nederlands Huisartsen Genootschap (NHG, Dutch College of General Practitioners) <https://www.nvk.nl/themas/kwaliteit/richtlijnen> , Nederlandse Vereniging voor Kindergeneeskunde (NVK, Dutch Association for Pediatrics) |
| Poland | <https://ginasthma.org/gina-reports/> , Global Strategy for Asthma Management and Prevention, Global Initiative for Asthma GINA |
| Italy | <https://ginasthma.org/gina-reports/> , Global Strategy for Asthma Management and Prevention, Global Initiative for Asthma GINA  Gestione dell’attacuto di asma in eta’ pediatrica, Linea Guida SIP - Aggiornamento 2016, Società Italiana di Pediatria (Italian Society of Pediatrics)  <https://www.brit-thoracic.org.uk/quality-improvement/guidelines/asthma/> , British Thoracic Society (BTC), Scottish Intercollegiate Guidelines Network (SIGN) |
| Germany | [www.awmf.org/leitlinien/detail/ll/nvl-002.html](http://www.awmf.org/leitlinien/detail/ll/nvl-002.html) , Arbeitsgemeinschaft der Wissenschaftlichen Medizinischen Fachgesellschaften (Working group of scientific medical societies)  <https://www.gpau.de/service/paediatrische-leitlinien/> , Gesellschaft für Pädiatrische Allergologie und Umweltmedizin (Society for Pediatric Allergology and Environmental Medicine) |
| Cyprus | <https://ginasthma.org/gina-reports/> , Global Strategy for Asthma Management and Prevention, Global Initiative for Asthma GINA |
